# Health Plan Switching and Health Care Utilization: A Randomized Clinical Trial

**DOI:** 10.1001/jamahealthforum.2024.0324

**Published:** 2024-03-29

**Authors:** Marina Lovchikova, Andrew Feher, Langou Lian

**Affiliations:** 1 Covered California, Sacramento; 2 Department of Health Care Access and Information, Sacramento, California

## Abstract

**Importance:**

While the Patient Protection and Affordable Care Act (ACA) helped make health insurance premiums more affordable with premium tax credits, ACA marketplace enrollees continue to face barriers to care.

**Objective:**

To investigate the effect of informational emails on plan switching and health care utilization.

**Design, Setting, and Participants:**

This randomized clinical trial was conducted during the 2021 special enrollment period in California’s Affordable Care Act marketplace among households that reported receiving unemployment insurance and were enrolled in non–silver-tier plans. The trial targeted 42 470 households that became temporarily eligible for cost-sharing reduction (CSR) silver plans that covered 94% of medical costs (CSR silver 94 plans) as a result of the 2021 American Rescue Plan Act.

**Intervention:**

Households were randomized to either a no-email control group or to a treatment group receiving 2 informational emails encouraging households to switch to CSR plans.

**Main Outcomes and Measures:**

The primary outcome was the switch rate to a CSR silver plan by July 31, 2021. Secondary outcomes include various measures of health care utilization in the second half of 2021 (July 1, 2021, to December 31, 2021). Health care utilization was measured by rates of practitioner visits, emergency department visits, hospitalizations, and prescription fills.

**Results:**

Of the 42 470 households (head of household mean [SD], age, 41.4 [13.3] years; 51.7% male), 10 650 (25.1%) were in the control group and 31 820 (74.9%) were in the treatment group. The emails led to a statistically significant 3.1–percentage point (95% CI, 2.6-3.6 percentage points) increase in CSR silver 94 enrollment (a 74.8% relative increase) by July 31, 2021, and a 1.3–percentage point (95% CI, 0.2-2.4 percentage points) increase (a 2.3% relative increase) in practitioner visits by December 31, 2021. The emails had no detectable effect on prescription fills, emergency department visits, or hospitalizations.

**Conclusions and Relevance:**

The results of this randomized clinical trial provide experimental evidence that, with access to more affordable health care, individuals are more likely to visit practitioners.

**Trial Registration:**

ClinicalTrials.gov Identifier: NCT05891418

## Key Points

**Question:**

What are the effects of informational emails on plan switching and health care utilization among households enrolled in Affordable Care Act marketplace coverage?

**Findings:**

In this randomized clinical trial that included 42 470 California households enrolled in non–silver-tier plans and eligible for plans that cover 94% of medical costs, informational emails led to a statistically significant 75% increase in switching to silver-tier plans.

**Meaning:**

These results show that policymakers can use low-cost emails to increase plan switching, which in turn can increase access to care.

## Teaser

This randomized clinical trial assesses whether email nudges during the 2021 special enrollment period in California’s Affordable Care Act marketplace led to increased enrollment in silver-tier health plans that cover 94% of medical costs and to increased health care utilization.

## Introduction

Since the implementation of the Patient Protection and Affordable Care Act (ACA) in 2014, uninsured rates have reached record lows,^1^ but many insured individuals face significant out-of-pocket expenses when accessing care, a problem that has only worsened in recent years. In 2019, for example, 33% of individuals in the US delayed medical treatment due to cost, and by 2022, this share increased to 38%.^2^ During the COVID-19 pandemic, policymakers passed a variety of policies to reduce barriers to care, but to date, little is known about their effects. We sought to fill this gap by reporting results from a randomized clinical trial during the 2021 special enrollment period in California’s ACA marketplace following passage of the American Rescue Plan Act (ARPA).

The ACA marketplaces feature 5 coverage options, known as metal tiers, that vary in the percentage of medical expenses covered: catastrophic (below 60%), bronze (60%), silver (70%), gold (80%), and platinum (90%). For the lowest-income consumers with incomes below 150% of the federal poverty level (ie, below $19 100 for an individual) who enroll in silver-tier coverage, the ACA offers eligibility for cost-sharing reduction (CSR) silver 94 plans that cover 94% of medical costs by substantially reducing deductibles, copayments, coinsurance, and out-of-pocket maximums. To help those affected by job loss, the American Rescue Plan Act (ARPA) temporarily expanded CSR silver 94 eligibility for the last 6 months of 2021 to all households receiving unemployment insurance regardless of income.

Importantly, though, eligibility for CSR silver 94 coverage does not automatically result in enrollment in a CSR silver 94 plan, and every year hundreds of thousands of silver 94–eligible individuals enroll in non–silver-tier plans.^3^ When forgoing CSR silver 94 coverage, these individuals miss out on reduced out-of-pocket expenses when accessing care, as well as much greater financial protection if an adverse health event should occur. For example, in 2021, Covered California enrollees in a silver 94 plan paid $5 for a primary care visit, paid $3 for a generic prescription drug fill, and had an out-of-pocket maximum of $1000; by contrast, Covered California enrollees in a bronze plan paid $65 for a primary care visit, paid $18 for a generic prescription drug fill, and had an out-of-pocket maximum of $8200 (see eAppendix 1 in Supplement 1 for additional details on 2021 Covered California benefit designs).

Drawing on a unique administrative enrollment and claims dataset, we used a randomized clinical trial to test the effects of informational emails on plan switching into CSR silver 94 coverage and health care utilization among 42 470 households that reported unemployment insurance as an income source and were enrolled in non–silver-tier plans. To the extent informational emails increase CSR silver 94 enrollment and access to care, they could be a valuable tool for ACA marketplace administrators.

## Methods

This randomized clinical trial was approved by the State of California Health and Human Services institutional review board, which did not require informed consent. The trial protocol and statistical analysis plan are provided in Supplement 2. The study followed the Consolidated Standards of Reporting Trials (CONSORT) reporting guideline.

### Participants and Study Design

We created a pool of study participants (households) in the last week of June 2021 after Covered California implemented the ARPA’s unemployment insurance provision to expand CSR silver 94 eligibility to households that reported unemployment insurance as an income source on their 2021 application. Households were included in the study pool if they were enrolled in non–silver-tier plans through Covered California on June 28, 2021, reported unemployment insurance as an income source, and provided an email address on their application. By conducting a randomized clinical trial immediately after implementation, we aimed to maximize the number of households eligible for the intervention while also leaving enough time for consumers to use their new plan if they switched.

**Figure 1.**
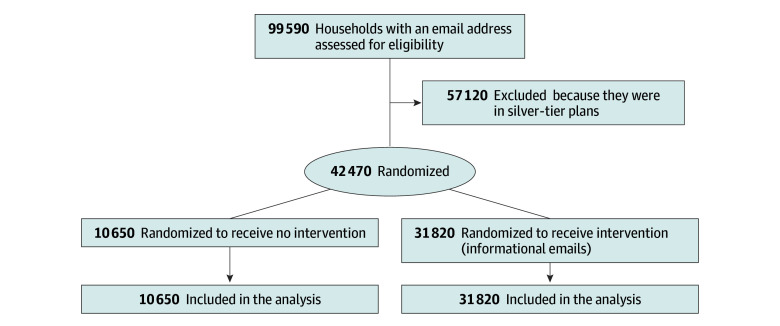
CONSORT Flow Diagram

In this study, we randomly assigned households to 1 of 2 groups: a control group and a treatment group. Of the 42 470 households, 25.1% were assigned to the control group, while the remaining 74.9% were assigned to the treatment group receiving emails (Figure 1). Randomization was performed by the second study author (A.F.) using Stata SE, version 16 (StataCorp LLC). We allocated a larger share of households to the treatment group to treat as many consumers as possible, while also learning about the effects of the intervention. Emails were sent in English but included a button at the top of the page to translate the content to Spanish from within the email.

Households in the control group received the required eligibility determination notice with the header “Important news about your health benefits”; the notice explained that Covered California reran their eligibility to apply additional financial help as part of the ARPA’s unemployment insurance provision and included information about cost sharing reductions to lower costs when accessing care (see eAppendix 2 in Supplement 1 for the intervention materials). Households in the treatment group received the required eligibility determination notice and were assigned to receive 2 informational emails in late June and mid-July. The email subject line was “You may save money by switching to a Silver 94 health plan for as low as $1 per month.” The emails contained premium information (“pay only $1 per month”), a list of copays for core health services from the CSR silver 94 standardized benefit design ($5 for primary practitioner visit, $3 for generic prescriptions, and a $75 annual deductible), and details on different ways to switch plans, such as through self-service online or working with an enrollment assister. Additionally, the email contained peer comparison language, noting that “90% of people who qualify for a CSR silver 94 choose it as their health plan.” Intervention materials cost approximately $0.01 per household for those assigned to the treatment group.

### Data Sources and Primary Outcome

We used administrative data from Covered California that provide household-level demographic, eligibility, enrollment, and health care utilization details for the 2021 enrollment year. Our primary outcome was an indicator for whether a household switched from a non–silver-tier to a CSR silver 94 plan by July 31, 2021. The administrative data also included pretreatment characteristics of households that we used to assess the validity of the random assignment: self-reported race and ethnicity of head of household, baseline metal tier, age of the head of household, and income as a percentage of federal poverty level (Table 1). Categories of race and ethnicity included Asian, Black, Hispanic or Latino, White, and other (all other races; these were combined because more than 10 were reported) or unknown (if the applicant did not choose to report it during the application).

**Table 1.** Characteristics of Study Participants at Baseline^a^

Characteristic	Households, No. (%)	
	Control group (n = 10 650)	Treatment group (n = 31 820)
Age of head of household, mean (SD), y	41.4 (13.3)	41.4 (13.2)
Sex		
Female	5095 (47.8)	15 424 (48.5)
Male	5555 (52.2)	16 396 (51.5)
Race and ethnicity of head of household		
Asian	1461 (13.7)	4458 (14.0)
Black	498 (4.7)	1460 (4.6)
Hispanic or Latino	2295 (21.5)	6708 (21.1)
White	3964 (37.2)	11982 (37.7)
Other or unknown^b^	2432 (22.8)	7212 (22.7)
Language preference of head of household		
Spanish	328 (3.1)	958 (3.0)
English	10 108 (94.9)	30 196 (94.9)
Health plan^c^		
Catastrophic	197 (1.8)	684 (2.1)
Bronze	7262 (68.2)	21 556 (67.7)
Gold	2095 (19.7)	6528 (20.5)
Platinum	1096 (10.3)	3052 (9.6)
Health care utilization^d^		
Practitioner visit	6038 (56.7)	17 998 (56.6)
Prescription fill	5934 (55.7)	17 615 (55.4)
Emergency department visit	641 (6.0)	1880 (5.9)
Hospitalization	170 (1.6)	543 (1.7)
Subsidy ineligible	365 (3.4)	1030 (3.2)
Percentage of federal poverty level, mean (SD)	242.3 (130.6)	242.2 (135.8)

^a^ Self-reported by the head of household in application for 2021 coverage year submitted before June 30, 2021.

^b^ Included all other races (these were combined because more than 10 were reported) and unknown race if applicant did not choose to report it during the application.

^c^ Plan level in which household was enrolled at baseline.

^d^ Includes use of health care service in the pretreatment period (between January 1, 2021, and June 30, 2021).

Our secondary outcomes were indicators of health care utilization, including whether anyone enrolled in the household had a practitioner visit, emergency department visit, hospitalization, or prescription drug fill in the last 6 months of 2021. The full definitions for these measures are provided in eAppendix 3 in Supplement 1. Utilization measures were summarized at the household level for the pretreatment (January 1, 2021, to June 30, 2021) and posttreatment (July 1, 2021, to December 31, 2021) periods.

For all primary and secondary outcomes, we conducted prespecified subgroup analyses based on the pretreatment household characteristics listed above (Table 2, Table 3, and eAppendix 4 in Supplement 1, ). Additionally, we conducted an exploratory subgroup analysis, stratifying the sample by pretreatment utilization, and examined the probability of staying in a CSR silver 94 plan until December 31, 2021 (eAppendix 5 in Supplement 1).

**Table 2.** Absolute and Relative Impact of Email Nudges on Probability of Practitioner Visits, by Pretreatment Characteristics^a^

Subgroup	Households, No.	Households with at least 1 practitioner visit, No. (visit probability, %)		Absolute impact of email nudges, percentage points (95% CI)	Relative impact of email nudges, %
		Control group	Treatment group		
All	42 470	6052 (56.8)	18 500 (58.1)	1.3 (0.2 to 2.4)	2.3
Age of head of household, y					
≤30	10 768	1379 (51.3)	4195 (51.9)	0.6 (−1.6 to 2.8)	1.2
31-49	17 966	2453 (54.9)	7675 (56.9)	1.9 (0.2 to 3.6)	3.5
≥50	13 736	2220 (63.5)	6630 (64.7)	1.2 (−0.6 to 3.1)	2.0
Race and ethnicity of head of household					
Asian	5919	756 (51.7)	2369 (53.1)	1.4 (−1.6 to 4.3)	2.7
Black	1958	271 (54.4)	851 (58.3)	3.9 (−1.2 to 8.9)	7.1
Hispanic or Latino	9003	1262 (55.0)	3762 (56.1)	1.1 (−1.3 to 3.5)	2.0
White	15 946	2338 (59.0)	7265 (60.6)	1.7 (−0.1 to 3.4)	2.8
Other or unknown^b^	9644	1425 (58.6)	4253 (59.0)	0.4 (−1.9 to 2.6)	0.6
Federal poverty level, %					
≤200	21 258	2731 (51.0)	8311 (52.3)	1.3 (−0.2 to 2.9)	2.6
201-400	18 094	2761 (61.5)	8615 (63.3)	1.8 (0.1 to 3.4)	2.9
≥401	3118	560 (69.7)	1574 (68.0)	−1.6 (−5.3 to 2.1)	−2.3
Health plan^c^					
Catastrophic	881	74 (37.6)	284 (41.5)	4.0 (−3.8 to 11.7)	10.5
Bronze	28 818	3693 (50.9)	11 216 (52.0)	1.2 (−0.2 to 2.5)	2.3
Gold	8623	1443 (68.9)	4666 (71.5)	2.6 (0.3 to 4.9)	3.8
Platinum	4148	842 (76.8)	2334 (76.5)	−0.4 (−3.3 to 2.6)	−0.5

^a^ Table reports intent-to-treat estimates.

^b^ Included all other races (these were combined because more than 10 were reported) and unknown race if individuals opted not to provide a specific race or ethnicity when applying.

^c^ Plan level in which household was enrolled at baseline.

**Table 3.** Absolute and Relative Plan Switch Rates to Silver-Tier Plans, by Consumer Pretreatment Characteristics^a^

Characteristic	Households, No.	Plan switchers, No. (switch rate, %)		Absolute impact of email nudges, percentage points (95% CI)	Relative impact of email nudges, %
		Control group	Treatment group		
All	42 470	441 (4.1)	2303 (7.2)	3.1 (2.6-3.6)	74.8
Age of head of household, y					
≤30	10 768	83 (3.1)	454 (5.6)	2.5 (1.7-3.4)	82.0
31-49	17 966	173 (3.9)	862 (6.4)	2.5 (1.8-3.2)	64.8
≥50	13 736	185 (5.3)	987 (9.6)	4.3 (3.4-5.3)	82.1
Federal poverty level, %					
≤200	21 258	134 (2.5)	719 (4.5)	2.0 (1.5-2.6)	80.9
201-400	18 094	246 (5.5)	1300 (9.6)	4.1 (3.2-4.9)	74.2
≥401	3118	61 (7.6)	284 (12.3)	4.7 (2.4-7.0)	61.8
Race and ethnicity of head of household					
Asian	5919	70 (4.8)	414 (9.3)	4.5 (3.1-5.9)	93.8
Black	1958	14 (2.8)	90 (6.2)	3.4 (1.4-5.3)	119.3
Hispanic or Latino	9003	73 (3.2)	386 (5.8)	2.6 (1.7-3.5)	80.9
White	15 946	169 (4.3)	826 (6.9)	2.6 (1.9-3.4)	61.7
Other or unknown^b^	9644	115 (4.7)	587 (8.1)	3.4 (2.4-4.5)	72.1
Health plan^c^					
Catastrophic	881	7 (3.6)	47 (6.9)	3.3 (0.1-6.5)	93.4
Bronze	28 818	342 (4.7)	1790 (8.3)	3.6 (3.0-4.2)	76.3
Gold	8623	70 (3.3)	346 (5.3)	2.0 (1.0-2.9)	58.6
Platinum	4148	22 (2)	120 (3.9)	1.9 (0.8-3.0)	95.9

^a^ Table reports intent-to-treat estimates.

^b^ Included all other races (these were combined because more than 10 were reported) and unknown race if individuals opted not to provide a specific race or ethnicity when applying.

^c^ Plan level in which household was enrolled at baseline.

### Statistical Analysis

Analysis was conducted in Stata, version 18 (StataCorp LLC). To estimate the effect of the email nudges, we used linear regression models with robust SEs to account for heteroskedasticity. This intent-to-treat analysis provides an estimate of the causal effect of being assigned to the informational emails. In addition to our primary specification, we used 2-stage least squares regression analysis to estimate complier average causal effects, which provide insight into the effects of CSR silver coverage on utilization among households induced to switch to CSR silver plans as a result of random assignment. We report those results in eAppendix 6 in Supplement 1. Statistical significance was defined as a 2-sided *P* < .05.

## Results

**Figure 2.**
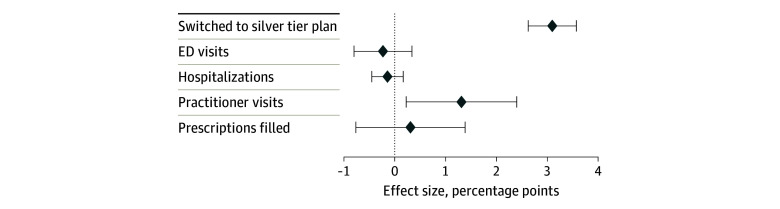
Intention-to-Treat Estimates of the Effect of Email Nudges Diamonds represent outcomes; whiskers, 95% CIs. Practitioner visits included any services received in an office setting or via telehealth visits other than patient’s home. ED indicates emergency department.

Of the 42 470 households (head of household mean [SD], age, 41.4 [13.3] years; 48.3% female and 51.7% male), 10 650 (25.1%) were in the control group and 31 820 (74.9%) were in the treatment group (Table 1). By July 31, 2021, 2303 households in the treatment group (7.2%) had switched from a non–silver-tier to a CSR silver 94 plan compared with 441 households in the control group (4.1%). Relative to the control group, assignment to email nudges increased the CSR silver 94 enrollment rate by a statistically significant 3.1 percentage points (95% CI, 2.6-3.6 percentage points; *P* < .001), which represents a 74.8% increase above the control group mean and amounted to 986 marginal switchers (Figure 2). For secondary outcomes, we found a statistically significant 1.3 percentage point increase in the probability of practitioner visits (95% CI, 0.2 to 2.4 percentage points [a 2.3% relative increase]) in the last 6 months of 2021 (Table 2). We also found that assignment to the treatment group decreased emergency room visits (−0.2 percentage points; 95% CI, −0.8 to 0.3) and hospitalizations (−0.1 percentage points; 95% CI, −0.4 to 0.2 ) and increased prescription drug fills (0.3 percentage points; 95% CI, −0.8 to 1.4), but those estimates are imprecisely estimated (eAppendix 4 in Supplement 1).

In prespecified subgroup analyses of CSR silver 94 enrollment, we detected significant differences in the effect of email nudges across most subgroups. The smallest absolute effect was among those in platinum plans (1.9 percentage points [95% CI, 0.8-3.0 percentage points], or a 95.9% increase relative to control group), and the largest absolute effect was among those with incomes above 400% of the federal poverty level (4.7 percentage points [95% CI, 2.4-7.0 percentage points], or a 61.8% relative increase). In examining effects by self-reported race and ethnicity, in absolute terms, Hispanic enrollees were least responsive to the email nudge (2.6 percentage points [95% CI, 1.7-3.5 percentage points], or an 80.9% relative increase), while Asian enrollees were most responsive (4.5 percentage points [95% CI, 3.1-5.9 percentage points], or 93.8%) (Table 3).

Similarly, we conducted prespecified subgroup analyses for health care utilization measures. The group of 31- to 49-year-olds increased practitioner visits by 1.9 percentage points (95% CI, 0.2-3.6 percentage points; a 3.5% increase relative to control group), and households with incomes between 201% and 400% of the federal poverty level increased practitioner visits by 1.8 percentage points (95% CI, 0.1-3.4 percentage points; a 2.9% relative increase). The largest absolute increase in rates of practitioner visits was among households in gold-tier plans at baseline (2.6 percentage points [95% CI, 0.3 to 4.9 percentage points], or a 3.8% relative increase) (Table 2). For emergency department visits, hospitalizations, and prescription fills, there were no statistically significant subgroup differences (eAppendix 4 in Supplement 1).

## Discussion

CSR silver plans are designed to reduce barriers to care for ACA marketplace enrollees. But hundreds of thousands of consumers eligible for these plans select catastrophic, bronze, gold, or platinum plans instead, often paying substantially higher premiums and out-of-pocket expenses.^4^ Following passage of the American Rescue Plan Act during the 2021 special enrollment period in California’s ACA marketplace, we conducted a randomized clinical trial and found that low-cost email nudges significantly increased CSR silver 94 enrollment and practitioner visits among unemployment insurance recipients enrolled in non–silver-tier plans. The emails did not, however, detectably affect prescription drug use, emergency department visits or hospitalizations.

While the intervention induced nearly 1000 households to switch to CSR silver 94 plans for the last 6 months of 2021, over 90% of households remained in non–silver-tier plans. That high rate of inertia is consistent with past studies wherein enrollees did not switch plans even when they stood to reap substantial benefits.^5,6,7,8,9,10,11,12^ In the absence of automatically defaulting eligible consumers into CSR silver 94 plans, marketplaces will need to continue to test and refine how they communicate the benefits these plans confer.

In terms of utilization, our results align with the canonical RAND Health Insurance Experiment that found that use of health care services increased with more generous cost sharing.^13^ Here, too, while we see an encouraging pattern of utilization with an increase in low-cost practitioner visits and decreases in high-cost emergency department visits and hospitalizations, our observed effects are small. For that reason, future researchers seeking to study the downstream utilization of marketplace enrollees will need large sample sizes, stronger interventions, and preferably both.^14^

### Limitations

While our research design was based on random assignment, which provides a strong basis for causal inference, our study has some limitations. First, the ARPA’s unemployment insurance provision was only in effect for the last 6 months of 2021; it is possible that access to more affordable coverage for a longer period of time could have had different effects.^15^ Second, our measure of practitioner visits only partially includes telehealth, omitting telehealth visits at a patient’s home. Given that telehealth appointments significantly increased during the COVID-19 pandemic,^16^ we potentially underestimated the effect on health care utilization.

Third, the low rate of plan switching in our sample (less than 8%) makes it difficult to detect large changes in health care utilization, especially for low-probability events like emergency department visits and hospitalizations. This limitation is even more pronounced in subgroup analyses, which require even larger point estimates to detect statistically significant effects.

Additionally, our intervention took place in California, which has standardized benefit designs that allow consumers to compare plans based on the price, network, and quality ratings of the qualified health plans without the added complication of having to compare varying deductibles or copays across plans within the same metal tier. In states with more variable benefit designs, the effects of plan switching in general and in the context of the ARPA unemployment provision might differ.

Finally, our study occurred in a unique policy context during the COVID-19 pandemic among a subpopulation of unemployment insurance recipients and might not generalize to other health insurance settings. However, in light of recent state-level initiatives in New Mexico, Massachusetts, and California to broaden access to CSR silver 94–level coverage beyond 150% of the federal poverty level, we believe our results can inform ongoing efforts to improve affordability on the ACA marketplaces.^17,18,19^

## Conclusion

This randomized clinical trial found that email nudges led to statistically significant increases in CSR silver 94 enrollment and in practitioner visits but had no detectable effect on prescription drug use, emergency department visits, or hospitalizations. Thus, these results have implications for ACA marketplace administrators seeking effective ways of helping consumers select the best available plan for which they are eligible, which in turn can improve access to care and use of low-cost services.

## Notes

Supplement 1. **eAppendix 1.** 2021 Covered California benefit designs **eAppendix 2.** Intervention materials **eAppendix 3.** Definitions of health care utilization measures used in the study **eAppendix 4.** Pre-specified subgroup analysis (intent-to-treat estimates) **eAppendix 5.** Exploratory analysis (intent-to-treat) **eAppendix 6.** Complier Average Causal Effect (CACE) estimates for primary and secondary outcomes

Supplement 2.
Trial Protocol and Statistical Analysis Plan

Supplement 3.
Data Sharing Statement
