# Supplementary material for: Health Plan Switching and Health Care Utilization: A Randomized Clinical Trial
Source: JAMA Health Forum. 2024 Mar 29;5(3):e240324. doi: 10.1001/jamahealthforum.2024.0324 (PMC10980954; doi:10.1001/jamahealthforum.2024.0324)
Supplement: Supplement 1. — eAppendix 1. 2021 Covered California benefit designs eAppendix 2. Intervention materials eAppendix 3. Definitions of health care utilization measures used in the study eAppendix 4. Pre-specified subgroup analysis (intent-to-treat estimates) eAppendix 5. Exploratory analysis (intent-to-treat) eAppendix 6. Complier Average Causal Effect (CACE) estimates for primary and secondary outcomes [file jamahealthforum-e240324-s001.pdf]

Supplemental Online Content

Lovchikova M, Feher A, Lian L. Health plan switching and health care utilization: a randomized clinical trial. *JAMA Health Forum*. 2024;5(3):e240324. doi:10.1001/jamahealthforum.2024.0324

eAppendix 1. 2021 Covered California benefit designs

eAppendix 2. Intervention materials

eAppendix 3. Definitions of health care utilization measures used in the study

eAppendix 4. Pre-specified subgroup analysis (intent-to-treat estimates)

eAppendix 5. Exploratory analysis (intent-to-treat)

eAppendix 6. Complier Average Causal Effect (CACE) estimates for primary and secondary outcomes

This supplemental material has been provided by the authors to give readers additional information about their work.

© 2024 Lovchikova M et al. *JAMA Health Forum.*

eAppendix 1. 2021 Covered California Benefit Designs

Covered California requires qualified health plans to offer standardized benefit designs in which all plans sold on the individual market in the same metal tier have the same cost-sharing and deductible profiles. This in turn enables consumers to compare plans based on the price, network, and quality ratings of the qualified health plans without the added complication of having to understand and compare varying deductibles or copays across plans within the same metal tier.

COVERED CALIFORNIA™

2021 Patient-Centered Benefit Designs and Medical Cost Shares

Benefits in blue are NOT subject to a deductible. Benefits in blue with a white corner are subject to a deductible after the first three visits.

Coverage Category	Minimum Coverage	Bronze	Silver	Enhanced Silver 73	Enhanced Silver 87	Enhanced Silver 94	Gold	Platinum
Percent of cost coverage	Covers **0%** until out-of-pocket maximum is met	Covers **60%** average annual cost	Covers **70%** average annual cost	Covers **73%** average annual cost	Covers **87%** average annual cost	Covers **94%** average annual cost	Covers **80%** average annual cost	Covers **90%** average annual cost
Cost-sharing Reduction Single Income Range	N/A	N/A	N/A	**$25,521 to $31,900 (>200% to ≤250% FPL)**	**$19,141 to $25,520 (>150% to ≤200% FPL)**	**up to $19,140 (100% to ≤150% FPL)**	N/A	N/A
Annual Wellness Exam	$0	$0	$0	$0	$0	$0	$0	$0
Primary Care Vist	After first 3 non-preventive visits, full cost per instance until out-of-pocket maximum is met	$65*	$40	$35	$15	$5	$35	$15
Urgent Care		$65*	$40	$35	$15	$5	$35	$15
Specialist Visit	Full cost per service until out-of-pocket maximum is met	$95*	$80	$75	$25	$8	$65	$30
Emergency Room Facility		40% after deductible is met	$400	$400	$150	$50	$350	$150
Laboratory Tests		$40	$40	$40	$20	$8	$40	$15
X-Rays and Diagnostics		40% after deductible is met	$85	$85	$40	$8	$75	$30
Imaging			$325	$325	$100	$50	$150 copay or 20% coinsurance***	$75 copay or 10% coinsurance***
Tier 1 (Generic Drugs)	Full cost per script until out-of-pocket maximum is met	$18**	$16**	$16**	$5 or less	$3 or less	$15 or less	$5 or less
Tier 2 (Preferred Drugs)		40% up to $500 after drug deductible is met	$60**	$55**	$25**	$10 or less	$55 or less	$15 or less
Tier 3 (Non-preferred Drugs)			$90**	$85**	$45**	$15 or less	$80 or less	$25 or less
Tier 4 (Specialty Drugs)			20% up to $250** per script	20% up to $250** per script	15% up to $150** per script	10% up to $150 per script	20% up to $250 per script	10% up to $250 per script
Medical Deductible	N/A	Individual: $6,300 Family: $12,600	Individual: $4,000 Family: $8,000	Individual: $3,700 Family: $7,400	Individual: $1,400 Family: $2,800	Individual: $75 Family: $150	N/A	N/A
Pharmacy Deductible	N/A	Individual: $500 Family: $1,000	Individual: $300 Family: $600	Individual: $275 Family: $550	Individual: $100 Family: $200	N/A	N/A	N/A
Annual Out-of-Pocket Maximum	$8,150 individual only	$8,200 individual $16,400 family	$8,200 individual $16,400 family	$6,500 individual $13,000 family	$2,850 individual $5,700 family	$1,000 individual $2,000 family	$8,200 individual $16,400 family	$4,500 individual $9,000 family

Drug prices are for a 30 day supply.
* Copay is for any combination of services (primary care, specialist, urgent care) for the first three visits. After three visits, future visits will be at full cost until the medical deductible is met.
** Price is after pharmacy deductible amount is met.
*** See plan Evidence of Coverage for imaging cost share.

Note: Available online at https://www.coveredca.com/pdfs/2021-Health-Benefits-table.pdf

© 2024 Lovchikova M et al. *JAMA Health Forum.*

eAppendix 2. Intervention Materials

A standard notice of eligibility determination mailed to both groups in our study population.

A standard notice of eligibility determination is sent to all consumers whose eligibility to Covered California, Medi-Cal or Cost-Sharing Reduction plans has changed. If a consumer is already enrolled in a health insurance plan, a notice will include the plan details, monthly premium, financial help (advanced premium tax credit) and net-of-subsidy premium.

Covered California
PO Box 989725
West Sacramento, CA 95798-9725

COVERED CALIFORNIA
Your destination for affordable healthcare, including Medi-Cal

[redacted]

Important news about your health benefits

06/27/2021 Case Number: [redacted]

> The American Rescue Plan (2021 federal stimulus package) provides additional financial help for consumers who received Unemployment Insurance benefits in 2021. Because someone in your household added Unemployment Insurance benefits, we checked to see if you qualify for more or new financial help beginning 07/01/2021. If you are enrolled in a health plan, we applied the maximum amount of financial help you qualify for. We have sent this information to your health insurance company to make updates to your bill, if needed.

Dear [redacted],

Thank you for choosing Covered California. Unless you told us not to, we checked to see if your household members qualify for:

- Free or low-cost Medi-Cal
- A private health insurance plan through Covered California
- Financial help (premium tax credits and cost-sharing reductions)

If someone in your household qualifies for a private plan through Covered California, their coverage is for benefit year **2021**. Medi-Cal coverage may start sooner. Look for the person's name below to see what they qualify for.

[redacted]

We recently received updated information about your household. Based on this information, we have now re-evaluated your eligibility and it is listed below.

Covered California Eligibility
You are eligible for health and dental plans through Covered California. Because your household reported Unemployment Insurance benefits for 2021, your household qualifies for up to $1,382.96 per month in federal premium tax credits.

You also qualify for cost-sharing reductions (CSR). If you enroll in a Silver plan you can save money

CCOE100 [redacted] 1

when you receive medical care. Cost-sharing reductions lower out-of-pocket costs, such as copays, coinsurance and deductibles.

You do not qualify for the California Premium Subsidy because you do not meet the income requirements for the program.

Medi-Cal Eligibility
You do not qualify for Medi-Cal because your income is above the limit of $2,004.00 per month.

[redacted]

We recently received updated information about your household. Based on this information, we have now re-evaluated your eligibility and it is listed below.

Covered California Eligibility
You are eligible for health and dental plans through Covered California. Because your household reported Unemployment Insurance benefits for 2021, your household qualifies for up to $1,382.96 per month in federal premium tax credits.

You also qualify for cost-sharing reductions (CSR). If you enroll in a Silver plan you can save money when you receive medical care. Cost-sharing reductions lower out-of-pocket costs, such as copays, coinsurance and deductibles.

You do not qualify for the California Premium Subsidy because you do not meet the income requirements for the program.

Medi-Cal Eligibility
You do not qualify for Medi-Cal because your income is above the limit of $2,004.00 per month.

Plan Selection summary

Thank you for choosing a plan through Covered California. Below is a summary of the plan(s) you selected and the financial help you qualify for as of 06/27/2021.

In the chart below, the "Monthly Premium" is the total cost of your plan. "Financial Help" is the state and federal subsidy amount that lowers your monthly premium if you qualify. **The "Net Premium" is the amount you pay per month.**

Name	Plan	Monthly Premium	Financial Help	Net Premium
[redacted] Start Date: 07/01/2021<br>[redacted] Start Date: 07/01/2021	Health Net - Bronze 60 PureCare HSP	$1,144.50	-$1,142.50	**$2.00**

If the financial help column says "N/A", this means you did not ask for help paying for your health plan, or the listed individual or plan is not eligible for financial help.

CCOE100 [redacted] 2

© 2024 Lovchikova M et al. *JAMA Health Forum.*

Email sent to the treatment group

An email notified recipients that the amount of financial help was recalculated based on their unemployment income reported in their application and prominently featured the CSR silver 94 plan copays, deductible and emphasized free preventive care.

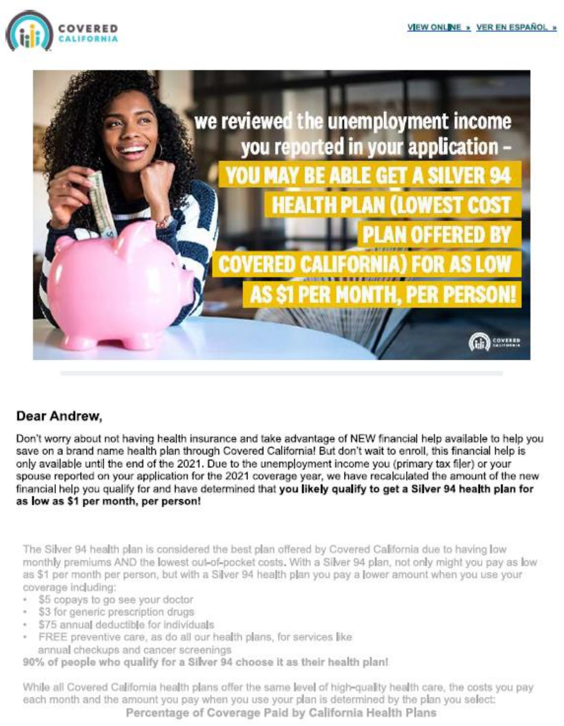

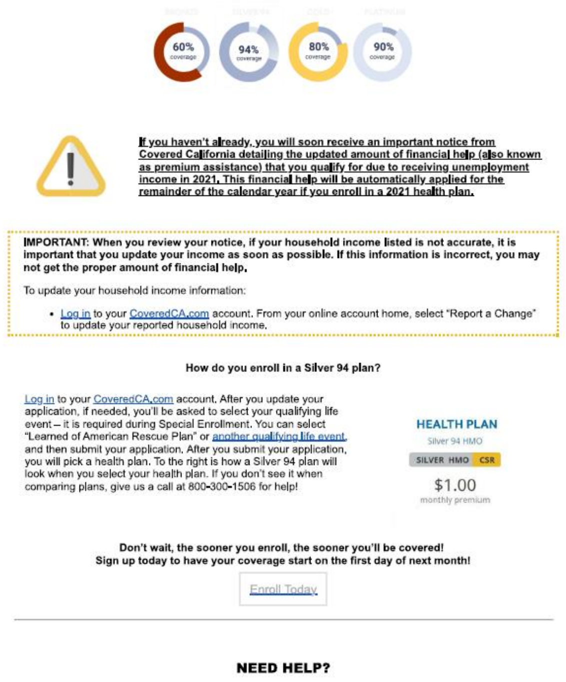

© 2024 Lovchikova M et al. *JAMA Health Forum.*

eAppendix 3. Definitions of health care utilization measures used in the study

Health Care Utilization Data: Covered California requires all contracted health plans to submit claims and utilization data as part of AB929.[1]

Measures used in the study are defined as follows:

Doctor Visits are professional visits provided in an office setting under medical coverage. The number of visits is based on the count of unique patient, service date and health care professionals ID combinations. An office setting is defined as a location other than a hospital, skilled nursing facility, military treatment facility, community health center, state or local public health clinic, or intermediate care facility. These are settings where health care professionals routinely provide health examinations, diagnosis and treatment of illness or injury on an ambulatory basis.

Prescriptions filled (excl. voided) Rx are prescriptions filled based on the RX Count field. The number of prescriptions filled is generally equal to the number of original or replacement pharmacy claims minus the number of voided pharmacy claims.

Emergency Room Visits are emergency room facility visits provided under medical coverage. The number of visits is based on the count of unique patient and service date combinations. This includes both ER visits that resulted in an admission and those that did not. Emergency room services identify all services provided in an emergency room setting or involving the activation of an emergency trauma team.

Hospitalizations (Acute Admissions) identify admissions that took place in an acute inpatient setting. Acute inpatient settings include inpatient hospitals, birthing centers, inpatient psychiatric facilities and residential substance abuse facilities.

---

[1] Assembly Bill 929 Paragraph 100503.7 (b) (1) states: “A qualified health plan shall provide data on enrollees to the Exchange in a form, manner, and frequency specified by the Exchange.” For additional details about legislation see https://leginfo.legislature.ca.gov/faces/billNavClient.xhtml?bill_id=201920200AB929

© 2024 Lovchikova M et al. *JAMA Health Forum.*

eAppendix 4. Pre-specified subgroup analysis (intent-to-treat estimates)

eTable 1. Effect of random assignment to email treatment group on prescriptions filled, emergency room visits and hospitalizations by consumer characteristics (intent-to-treat)

Outcome	Subgroup	N	Control group mean (%)	Impact of email nudges (percentage points, pp)	% change	Standard Error of effect (pp)	p-value
ER visits	**All**	**42,470**	**7.3**	**-0.2**	**-3.1**	**0.3**	**0.43**
	**Age**						
	Up To 30	10,768	6.8	0.2	2.5	0.6	0.76
	31-49	17,966	7.2	-0.4	-5.4	0.4	0.38
	50+	13,736	7.8	-0.3	-4.1	0.5	0.54
	**Federal poverty level (FPL), %**						
	Up To 200%	21,258	6.4	0.0	0.8	0.4	0.90
	200%-400%	18,094	8.0	-0.3	-3.3	0.5	0.57
	400% +	3,118	9.0	-2.0	-21.8	1.1	0.09
	**Race**						
	Asian	5,920	5.5	-1.4	-24.4	0.7	0.04
	Black	1,958	8.4	0.5	5.9	1.5	0.73
	Hispanic/ Latino	8,990	8.3	-0.6	-7.6	0.7	0.34
	White	15,986	7.3	-0.1	-1.1	0.5	0.87
	Other	3,972	6.7	1.0	14.4	0.9	0.30
	Unknown	5,644	7.3	0.2	2.5	0.8	0.81
	**Plan level**						
	Catastrophic	881	2.0	2.2	108.8	1.3	0.08
	Bronze	28,818	5.8	-0.3	-4.8	0.3	0.37
	Gold	8,623	10.2	-0.6	-5.7	0.8	0.44
	Platinum	4,148	12.4	0.6	4.6	1.2	0.63
Prescriptions filled (excl. voided)	**All**	**42,470**	**60.2**	**0.3**	**0.5**	**0.5**	**0.57**
	**Age**						
	Up To 30	10,768	51.1	1.1	2.2	1.1	0.31
	31-49	17,966	58.2	0.1	0.2	0.9	0.90
	50+	13,736	70.0	0.2	0.3	0.9	0.82
	**Federal poverty level (FPL), %**						
	Up To 200%	21,258	54.9	0.8	1.4	0.8	0.34
	200%-400%	18,094	64.6	0.2	0.3	0.8	0.84
	400% +	3,118	71.3	-2.1	-3.0	1.9	0.25
	**Race**						
	Asian	5,920	54.7	1.4	2.5	1.5	0.36
	Black	1,958	54.7	2.7	4.9	2.6	0.30
	Hispanic/ Latino	8,990	56.8	1.0	1.8	1.2	0.38
	White	15,986	64.8	-0.7	-1.0	0.9	0.44
	Other	3,972	59.7	0.8	1.4	1.8	0.65
	Unknown	5,644	60.8	-0.4	-0.7	1.5	0.76
	**Plan level**						
	Catastrophic	881	48.7	-1.4	-2.8	4.0	0.74
	Bronze	28,818	55.0	0.5	1.0	0.7	0.43
	Gold	8,623	71.6	0.6	0.8	1.1	0.62

© 2024 Lovchikova M et al. *JAMA Health Forum.*

	Platinum	4,148	75.3	-1.2	-1.6	1.5	0.42
Hospitalization	**All**	**42,470**	**2.0**	**-0.1**	**-6.8**	**0.2**	**0.38**
	**Age**						
	Up To 30	10,768	1.8	-0.3	-15.4	0.3	0.34
	31-49	17,966	2.0	0.1	2.5	0.2	0.83
	50+	13,736	2.3	-0.3	-12.3	0.3	0.33
	**Federal poverty level (FPL), %**						
	Up To 200%	21,258	1.6	-0.0	-0.0	0.2	1.00
	200%-400%	18,094	2.4	-0.2	-9.7	0.3	0.37
	400% +	3,118	3.1	-0.6	-18.0	0.7	0.42
	**Race**						
	Asian	5,920	1.3	0.2	15.4	0.3	0.56
	Black	1,958	2.2	-0.5	-22.3	0.7	0.51
	Hispanic/ Latino	8,990	1.9	-0.0	-0.5	0.3	0.98
	White	15,986	2.1	-0.1	-4.0	0.3	0.75
	Other	3,972	2.7	-0.5	-19.0	0.6	0.38
	Unknown	5,644	2.5	-0.5	-19.2	0.5	0.30
	**Plan level**						
	Catastrophic	881	0.0	0.4		0.3	0.08
	Bronze	28,818	1.3	-0.1	-3.9	0.2	0.74
	Gold	8,623	3.6	-0.5	-14.0	0.5	0.27
	Platinum	4,148	4.4	-0.1	-1.2	0.7	0.94

© 2024 Lovchikova M et al. *JAMA Health Forum.*

eAppendix 5. Exploratory analysis (intent-to-treat)

eTable 2. Exploratory subgroup analysis by pre-treatment utilization group for health care utilization measures (Intent-to-treat estimates)

**Outcome**	**Subgroup**	**N**	**Control group mean (%)**	**Impact of email nudges (percentage points, pp)**	**% change**	**Standard Error of effect (pp)**	**p-value**
ER visits	Had no service	39,949	6.5	-0.3	-4.0	0.3	0.36
	Had service	2,521	20.1	0.5	2.6	1.8	0.78
Doctor visits	Had no service	18,434	37.6	0.5	1.4	0.8	0.53
	Had service	24,036	71.5	2.0	2.8	0.7	0.003
Prescriptions filled (excl. voided)	Had no service	18,921	36.8	1.1	2.9	0.8	0.18
	Had service	23,549	78.9	-0.0	-0.0	0.6	0.96
Hospitalization	Had no service	41,757	1.8	-0.1	-6.1	0.1	0.45
	Had service	713	15.9	-2.6	-16.5	3.2	0.41

Note: Tables reports post-hoc subgroup analysis for health care utilization measures (intent-to-treat estimates). “Had no service” (“Had service”) indicates whether a household had a service defined in outcome column in pre-treatment period, e.g. with outcome “ER visits” the subgroup “Had no service” had not visited ER in pre-treatment period.

eTable 3. Exploratory analysis of the effect of email nudges on the duration of enrollment in CSR silver 94 plans

**Outcome**	**N**	**Control group mean (%)**	**Impact of email nudges (percentage points, pp)**	**% change**	**Standard Error of effect (pp)**	**p-value**
Enrolled in CSR Silver 94 plan in December 2021	42,470	5.6	2.8	49.4	0.3	<0.001

Note: Outcome variable is a binary indicator and equals 1 if household was enrolled in a CSR silver 94 plan in December 2021.

© 2024 Lovchikova M et al. *JAMA Health Forum.*

eAppendix 6. Complier Average Causal Effect (CACE) estimates for primary and secondary outcomes

In the main text, we report the intent-to-treat (ITT) effect – that is, the effect of treatment assignment on our outcomes of interest. But because only a small share of households assigned to the treatment group ended up switching to a CSR silver 94 plan (less than 8%), the effects of randomization to emails on utilization may be muted.

For that reason, we complement our ITT analysis with another causal estimand, the complier average causal effect (CACE). Below we use instrumental variable (IV) regression to estimate the CACE overall and by subgroup; this estimand provides insight into the effects of CSR silver coverage on utilization among households induced to switch to CSR silver plans as a result of random assignment. For the IV analysis to provide valid causal inferences, five assumptions must hold: (1) treatment assignment is random, (2) the Stable Unit Treatment Value Assumption (SUTVA), (3) the exclusion restriction (4) monotonicity and (5) treatment assignment must have a non-zero effect on the treatment.

The identifying assumptions are likely satisfied based on our experimental design, but we encourage readers to exercise caution when interpreting the CACE estimates due to concerns about the plausibility of the effect sizes. As the 95% confidence intervals in the table below show, for three of the four utilization outcomes, we are unable to rule out an impossibly large range of values. The point estimates are beset by a similar issue. As an illustrative example, recall that the control group's hospitalization rate was 2.0%, so a -4.5-percentage point CACE estimate implies a negative hospitalization probability.

eTable 4. Absolute probability of health care utilization (CACE estimates)

**Outcome**	**Effect (95% CI)**
ER visits	-7.4 (-25.8 to 11)
Hospitalization	-4.5 (-14.5 to 5.5)
Office visits	42.4 (7.3 to 77.5)
Prescriptions filled (excl. voided)	10.1 (-24.5 to 44.7)

© 2024 Lovchikova M et al. *JAMA Health Forum.*

eFigure1. Effect of CSR silver 94 enrollment on health care utilization (CACE)

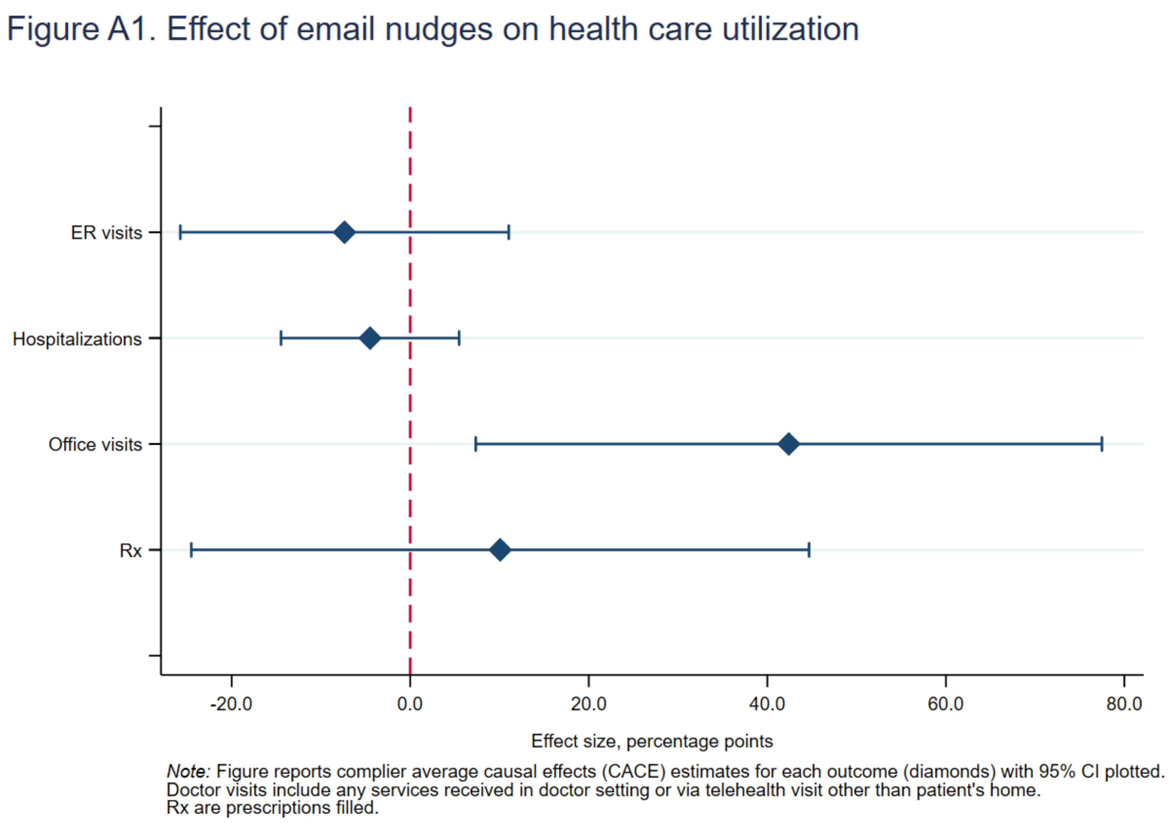

Note: Figure reports complier average causal effects (CACE) estimates for each outcome (diamonds) with 95% CI plotted. Doctor visits include any services received in office settings or via telehealth visit other than patient's home. Rx are prescription filled.

eTable 5. Absolute and relative probability of emergency room visits and doctor visits by consumer characteristics (CACE estimates)

Outcome	Subgroup	N	Control group mean (%)	Impact of email nudges (percentage points, pp)	% change	Standard Error of effect (pp)	p-value
ER visits	**All**	42,470	7.6	-7.4	-97.1	9.4	0.43
	**Age**						
	Up To 30	10,768	6.6	6.8	103.7	22.2	0.76
	31-49	17,966	7.8	-15.4	-198.2	17.8	0.39
	50+	13,736	8.2	-7.3	-89.9	12.1	0.54
	**Federal poverty level (FPL), %**						
	Up To 200%	21,258	6.4	2.4	37.6	19.2	0.90
	200%-400%	18,094	8.4	-6.5	-77.8	11.5	0.57
	400% +	3,118	12.1	-41.7	-344.1	26.2	0.11
	**Race**						
	Asian	5,920	6.9	-29.6	-428.3	15.4	0.05
	Black	1,958	8.0	15.6	195.9	46.2	0.74

© 2024 Lovchikova M et al. *JAMA Health Forum.*

	Hispanic/ Latino	8,990	9.1	-24.9	-272.4	26.8	0.35
	White	15,986	7.4	-3.0	-41.1	18.0	0.87
	Other	3,972	5.5	27.1	490.0	27.0	0.31
	Unknown	5,644	7.0	5.6	79.7	23.9	0.81
	**Plan level**						
	Catastrophic	881	-0.3	66.6	-19846.7	50.8	0.19
	Bronze	28,818	6.2	-7.8	-126.6	8.8	0.38
	Gold	8,623	11.2	-29.5	-264.4	39.4	0.45
	Platinum	4,148	11.8	29.4	249.0	61.2	0.63
Doctor visits	**All**	42,470	55.1	42.4	77.0	17.9	0.02
	**Age**						
	Up To 30	10,768	50.6	24.3	48.2	43.9	0.58
	31-49	17,966	52.0	76.7	147.6	35.0	0.03
	50+	13,736	62.0	28.6	46.2	21.6	0.18
	**Federal poverty level (FPL), %**						
	Up To 200%	21,258	49.3	65.5	132.9	39.5	0.10
	200%-400%	18,094	59.2	43.3	73.1	20.6	0.04
	400% +	3,118	72.3	-34.8	-48.1	42.2	0.41
	**Race**						
	Asian	5,920	50.3	30.2	59.9	32.8	0.36
	Black	1,958	52.6	91.0	173.2	86.1	0.29
	Hispanic/ Latino	8,990	53.0	54.5	102.8	47.8	0.25
	White	15,986	56.5	61.1	108.1	34.4	0.08
	Other	3,972	56.1	15.9	28.3	51.0	0.76
	Unknown	5,644	59.1	11.4	19.3	44.6	0.80
	**Plan level**						
	Catastrophic	881	33.3	119.3	357.9	131.4	0.36
	Bronze	28,818	49.3	32.8	66.5	18.7	0.08
	Gold	8,623	64.4	132.6	205.8	65.0	0.04
	Platinum	4,148	77.2	-18.2	-23.6	77.6	0.81
Prescriptions filled (excl. voided)	**All**	42,470	59.8	10.1	16.8	17.7	0.57
	**Age**						
	Up To 30	10,768	49.7	44.9	90.4	44.2	0.31
	31-49	17,966	58.0	4.3	7.5	33.9	0.90
	50+	13,736	69.7	4.6	6.6	20.6	0.82
	**Federal poverty level (FPL), %**						
	Up To 200%	21,258	54.0	37.3	69.1	39.0	0.34
	200%-400%	18,094	64.4	4.2	6.5	20.2	0.84
	400% +	3,118	74.7	-45.3	-60.7	42.0	0.28
	**Race**						
	Asian	5,920	53.4	30.0	56.2	32.8	0.36
	Black	1,958	52.3	84.2	160.9	85.7	0.33
	Hispanic/ Latino	8,990	55.5	41.4	74.5	47.3	0.38
	White	15,986	65.9	-25.7	-39.0	33.6	0.44
	Other	3,972	58.7	23.4	39.9	50.6	0.64
	Unknown	5,644	61.5	-13.4	-21.8	44.9	0.77
	**Plan level**						
	Catastrophic	881	50.2	-41.1	-81.8	124.0	0.74
	Bronze	28,818	54.3	14.9	27.5	18.7	0.43
	Gold	8,623	70.7	28.9	40.8	57.8	0.62

© 2024 Lovchikova M et al. *JAMA Health Forum.*

	Platinum	4,148	76.6	-63.6	-83.1	81.5	0.44
Hospitalization	**All**	42,470	2.2	-4.5	-201.5	5.1	0.38
	**Age**						
	Up To 30	10,768	2.1	-10.9	-513.5	11.6	0.35
	31-49	17,966	2.0	2.0	104.2	9.7	0.83
	50+	13,736	2.6	-6.4	-245.6	6.6	0.34
	**Federal poverty level (FPL), %**						
	Up To 200%	21,258	1.6	-0.0	-1.5	9.8	1.00
	200%-400%	18,094	2.7	-5.7	-211.4	6.4	0.37
	400% +	3,118	4.0	-11.9	-297.5	15.1	0.43
	**Race**						
	Asian	5,920	1.1	4.4	398.5	7.6	0.56
	Black	1,958	2.6	-15.5	-588.8	23.8	0.51
	Hispanic/ Latino	8,990	1.9	-0.4	-20.6	13.0	0.98
	White	15,986	2.2	-3.2	-142.8	9.9	0.75
	Other	3,972	3.4	-14.5	-429.0	16.8	0.39
	Unknown	5,644	3.2	-14.2	-446.7	14.1	0.31
	**Plan level**						
	Catastrophic	881	-0.5	13.2	-2814.3	10.1	0.19
	Bronze	28,818	1.4	-1.4	-103.2	4.3	0.74
	Gold	8,623	4.4	-25.6	-576.6	24.5	0.30
	Platinum	4,148	4.4	-2.8	-63.9	37.4	0.94

© 2024 Lovchikova M et al. *JAMA Health Forum.*
