# Supplementary material for: Health Plan Switching and Health Care Utilization: A Randomized Clinical Trial
Source: JAMA Health Forum. 2024 Mar 29;5(3):e240324. doi: 10.1001/jamahealthforum.2024.0324 (PMC10980954; doi:10.1001/jamahealthforum.2024.0324)
Supplement: Supplement 2. — Trial Protocol and Statistical Analysis Plan [file jamahealthforum-e240324-s002.pdf]

ClinicalTrials.gov Protocol Registration and Results System (PRS) Receipt
Release Date: June 2, 2023

ClinicalTrials.gov ID: NCT05891418

Study Identification

Unique Protocol ID: 022023

Brief Title: Effect of Email Nudges on Plan Switching and Healthcare Utilization Among Unemployment Insurance Recipients

Official Title: Effect of Email Nudges on Plan Switching and Healthcare Utilization Among Unemployment Insurance Recipients

Secondary IDs:

Study Status

Record Verification: June 2023

Overall Status: Completed

Study Start: June 25, 2021 [Actual]

Primary Completion: December 31, 2021 [Actual]

Study Completion: December 31, 2021 [Actual]

Sponsor/Collaborators

Sponsor: Office of Evaluation Sciences

Responsible Party: Principal Investigator
Investigator: Andrew Feher [dyokum]
Official Title: Research Scientist
Affiliation: Office of Evaluation Sciences

Collaborators:

Oversight

U.S. FDA-regulated Drug: No

U.S. FDA-regulated Device: No

U.S. FDA IND/IDE: No

Human Subjects Review: Board Status: Approved
Approval Number: 2022-213
Board Name: California Health and Human Services Institutional Review Board
Board Affiliation:
Phone: 916-651-5599
Email: CPHS@chhs.ca.gov
Address:

Data Monitoring: No

FDA Regulated Intervention: No

Study Description

Brief Summary: In March 2021, President Biden signed into law the American Rescue Plan Act of 2021 (ARP), a landmark federal economic relief and stimulus package designed to provide support to Americans hit by the economic recession brought about by the COVID-19 global pandemic. The law provides increased federal premium tax credits (PTC), and ensures that consumers will pay no more than 8.5 percent of household income on health insurance premiums in 2021 and 2022, if enrolled through an Affordable Care Act marketplace like Covered California.

The ARP also provides additional PTC and cost-sharing reduction (CSR) benefits to eligible marketplace enrollees who report receiving unemployment insurance benefits (UIB) for at least one week in 2021. Under the law, for 2021 only, Covered California consumers will have their household income level treated as if it were at 138.1 percent of the federal poverty level (FPL), regardless of their projected annual income, which will make them eligible for a Silver 94 plan, and which offers the greatest value on cost-sharing benefits.

But in order to access those cost-sharing benefits, households must be in silver tier plans, but over 40,000 were not. This project's goal is to assess the effects of an informational email on plan switching into Cost Sharing Reduction Silver plans and downstream healthcare utilization.

The project's research design is a randomized intervention among approximately 42,500 enrolled households with an email address in non-silver tier plans. The investigators randomly assigned to either two informational emails or to a no email control group. The investigators then collected administrative data to examine plan switching behavior and healthcare utilization among households in the study.

Detailed Description:

Conditions

Conditions: Health Behavior

Keywords: Affordable Care Act
randomized controlled trial
nudge
American Rescue Plan
unemployment insurance

Study Design

Study Type: Interventional

Primary Purpose: Other

Study Phase: N/A

Interventional Study Model: Parallel Assignment

Number of Arms: 2

Masking: Single (Participant)

Allocation: Randomized

Enrollment: 42470 [Actual]

Arms and Interventions

Arms	Assigned Interventions
Experimental: Control Group with a standard eligibility notice<br>Control group was assigned to receive no email outreach in June and July beyond an eligibility redetermination notice.	Behavioral: Standard notice of eligibility determination<br>No extra outreach during intervention period.
Experimental: Treatment group with a standard eligibility notice and assigned two email nudges<br>Assigned to receive an eligibility redetermination notice along with two informational emails about the benefits of Cost-Sharing Reduction Silver plans.	Behavioral: Two informational emails with plan information<br>Informational emails about the benefits of Cost-Sharing Reduction Silver plans.

Outcome Measures

Primary Outcome Measure:

1. Cost-Sharing Reduction Silver Enrollment
   Percent of households enrolled in a Cost-Sharing Reduction Silver Plan

   [Time Frame: 1 month]
2. Office Visit
   Percent of households with an office visit

   [Time Frame: 6 months]
3. Prescription drug use
   Percent of households with a prescription drug fill

   [Time Frame: 6 months]
4. Emergency room visit
   Percent of households with an emergency room visit

   [Time Frame: 6 months]
5. Hospitalization
   Percent of households with a hospitalization

   [Time Frame: 6 months]

Eligibility

Minimum Age: 0 Years

Maximum Age: 74 Years

Sex: All

Gender Based: No

Accepts Healthy Volunteers: Yes

Criteria: Inclusion Criteria:

- Has email address, in a non-silver plan and reported unemployment insurance in 2021

Exclusion Criteria:

- In a silver plan, no email address

Contacts/Locations

Central Contact Person: Andrew Feher, PhD
Telephone: 8184298860
Email: andrew.feher@covered.ca.gov

Central Contact Backup:

Study Officials: Andrew Feher, PhD
Study Principal Investigator
Covered California

Locations: **United States, California**
Covered California
Sacramento, California, United States, 95815
Contact: Andrew Feher, PhD andrew.feher@covered.ca.gov

IPDSharing

Plan to Share IPD: Yes
The researchers are committed to sharing as much data as possible for replication, consistent with the privacy and security requirements of the state agency that govern the re-disclosure of the data.

Supporting Information:
Study Protocol

Time Frame:
Data will be available with investigator support upon publication

Access Criteria:
Replication data can be accessed via request to the primary investigator upon publication.

URL:

References

Citations:

Links:

Available IPD/Information:

Documents

Statistical Analysis Plan
Document Date: May 23, 2023
Uploaded: 06/02/2023 12:21

Official Title of the Study: Examining the Effect of Informational Emails on Cost-Sharing Reduction (CSR) Silver 94 Enrollment and Utilization Among Unemployment Insurance Recipients

Date of the document creation: October 18, 2022

Date of the document update: May 23, 2023

Design Document and Analysis Plan

Project Name: Examining the Effect of Informational Emails on Cost-Sharing Reduction (CSR) Silver 94 Enrollment and Utilization Among Unemployment Insurance Recipients
Date of creation: October 18, 2022

Project Objective

Using informational emails to increase CSR Silver enrollment among unemployment recipients

Evaluation Design

Test Arms / Treatment Conditions:

This is a randomized intervention where households who reported receiving unemployment insurance (UI) in 2021, had an email address and were enrolled in non-silver tier plans as of June 2021 were assigned to one of two arms: (1) a control group or (2) an informational email treatment group. Households in the treatment arm were assigned to receive two emails in June and July 2021 that encourage switching metal tiers (from Catastrophic, Bronze, Gold or Platinum to CSR Silver).

Total Number of Observations:

N = 42,470 households eligible for Cost-Sharing Reduction (CSR) Silver 94 plans but enrolled in non-silver tier plans in late-June 2021.

Randomization / Assignment:

Randomization was done at the household level. 75% of households were randomly assigned to two informational emails in late June and mid-July, respectively, and the remaining 25% were randomly assigned to no outreach during the intervention period beyond an eligibility redetermination notice.

Power:

To arrive at an estimate for the minimum detectable effect (MDE) for our primary outcome, we assume a baseline metal tier switch rate of 10 percent. The intervention was powered at the 80% level to detect a 1 percentage point increase in the CSR Silver enrollment rate.

Meaningful Effect Size:

In previous RCTs designed to induce plan switching, we have observed intent-to-treat (ITT) effects between 0.7 to 3.9 percentage points. Given the low-cost nature of this nudge (i.e. approximately $0.02 per household), even a 1 percentage point increase in CSR Silver take-up would be meaningful.

Likely Effect Size:

Based on prior choice error nudges carried out by Covered California, we would expect to observe an ITT effect between 1-4 percentage points.

Data and Data Structure

Outcomes:

The primary outcome of interest will be an indicator for whether a consumer is enrolled in a CSR Silver plan by the end of July 2021. Our secondary outcomes will include (1) an indicator for whether someone had an office visit between July 2021 and December 2021 (2) an indicator for whether someone had an prescription drug fill between July 2021 and December 2021, (3) an indicator for whether someone had

an emergency room visit between July 2021 and December 2021, (4) an indicator for whether someone had a hospital admission between July 2021 and December 2021.

Data:
We will use Covered California administrative data to obtain enrollment outcomes and baseline demographics for our sample and Healthcare Evidence Initiative data to obtain utilization outcomes.

Quality Control Checks:
After carrying out the randomization, we checked for balance across several observable covariates (e.g. language spoken, self-reported race and ethnicity, baseline metal tier and income as a percent of the federal poverty level), which indicated there were no significant dissimilarities across treatment arms.

Statistical Models & Hypothesis Tests

This section describes the statistical models and hypothesis tests that will make up the analysis —including any follow-ups on effects in the main statistical model and any exploratory analyses that can be anticipated prior to analysis.

Statistical Models:
Intent-to-treat: to estimate treatment effects, our primary analysis will be an intent-to-treat (ITT) specification, examining the effect of treatment assignment. We will estimate the effect of the treatment using ordinary least squares (OLS) regression. That is, we will regress the outcome of interest (e.g. Silver enrollment) for household *i* on the treatment indicator:

$$outcome_i = \propto + \beta_1 Emails_i + \varepsilon_i$$

The coefficient β_1 will be the estimate of the causal effect of the intent to treat of informational emails.

Complier average causal effect: we expect some noncompliance among those households assigned to receive emails as they may have opted out of email communications from Covered California or provided an invalid email address. Thus, to augment our ITT analysis, we will also estimate treatment effects based on treatment receipt, using two-stage least squares regression (2SLS).

Follow-Up Analyses
We will examine treatment heterogeneity by self-reported race and ethnicity, baseline metal tier, age bracket and income bracket. In addition, as part of our complier average causal effect analysis, we will examine the effects of CSR Silver enrollment (among households induced to enroll in a CSR Silver plan as a result of random assignment) on our four utilization outcomes.

Inference Criteria, Including Any Adjustments for Multiple Comparisons:
We will not perform any corrections for multiple hypothesis testing, and we will use two-tailed tests with p-values <= 0.05 to denote statistically significant effects.
