# Supplementary material for: Health Plan Switching and Health Care Utilization: A Randomized Clinical Trial
Source: JAMA Health Forum. 2024 Mar 29;5(3):e240324. doi: 10.1001/jamahealthforum.2024.0324 (PMC10980954; doi:10.1001/jamahealthforum.2024.0324)
Supplement: Supplement 3. — Data Sharing Statement [file jamahealthforum-e240324-s003.pdf]

Data Sharing Statement

Lovchikova. Health Plan Switching and Health Care Utilization. *JAMA Health Forum*. Published March 29, 2024. doi:10.1001/jamahealthforum.2024.0324

Data
Data available: No
